# Supplementary material for: BST2, a Novel Inhibitory Receptor, Is Involved in NK Cell Cytotoxicity through Its Cytoplasmic Tail Domain
Source: Int J Mol Sci. 2022 Sep 27;23(19):11395. doi: 10.3390/ijms231911395 (PMC9570199; doi:10.3390/ijms231911395)
Supplement: Supplementary file 1 [file ijms-23-11395-s001.zip › ijms-1874256-supplementary.pdf]

Supplementary data

BST2, a novel inhibitory receptor, is involved in NK cell cytotoxicity through its cytoplasmic tail domain

Jinsoo Oh, Eunbi Yi, Soo Kyung Jeong, Sehoon Park and Se-Ho *

College of Life Science and Biotechnology, Korea University, 145 Anam-ro, Seongbuk-gu, Seoul 02841, Korea
* Correspondence: sehopark@korea.ac.kr; Tel.: +82-2-3290-3160

1. Supplementary figures

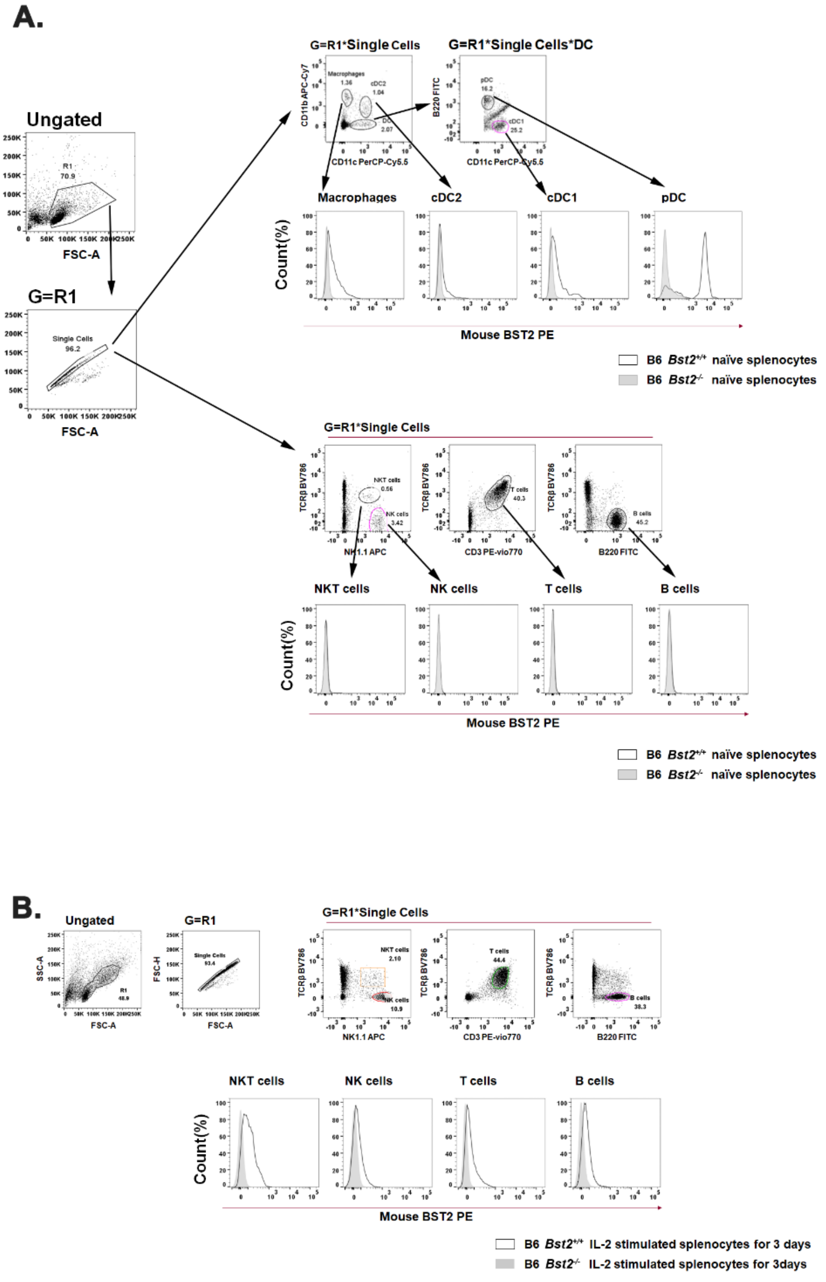

Figure S1. Lymphoid cells express BST2 after their activation. Naïve splenocytes of B6-origin *Bst2*$^{+/+}$ and *Bst2*$^{-/-}$ mice were stained with the following antibodies: anti-CD11b, anti-CD11c, anti-B220, anti-TCRβ, anti-NK1.1, anti-CD3, and anti-mouseBST2. (A) Myeloid cells were gated in detail and analyzed for their expression of BST2. Macrophages as CD11b^{+}CD11c^{-}; Conventional type 1 dendritic cells (cDC1) as CD11b^{-}CD11c^{high}B220^{-}; Conventional type 2 dendritic cells (cDC2) as CD11b^{+}CD11c^{+}; Plasmacytoid dendritic cells (pDC) as CD11b^{-}CD11c^{int}B220^{+}. Lymphoid cells were gated in detail and analyzed for their expression of BST2. NKT cells as TCRβ^{+}NK1.1^{+}; NK cells as TCRβ^{-}NK1.1^{+}; T cells as TCRβ^{+}CD3^{+}; B cells as TCRβ^{-}B220^{+}. (B) Naïve splenocytes of B6-origin *Bst2*$^{+/+}$ and *Bst2*$^{-/-}$ mice were stimulated by IL-2 treatment for three days. Activated lymphoid cells were gated with the same strategy described above and analyzed for their expression of BST2.

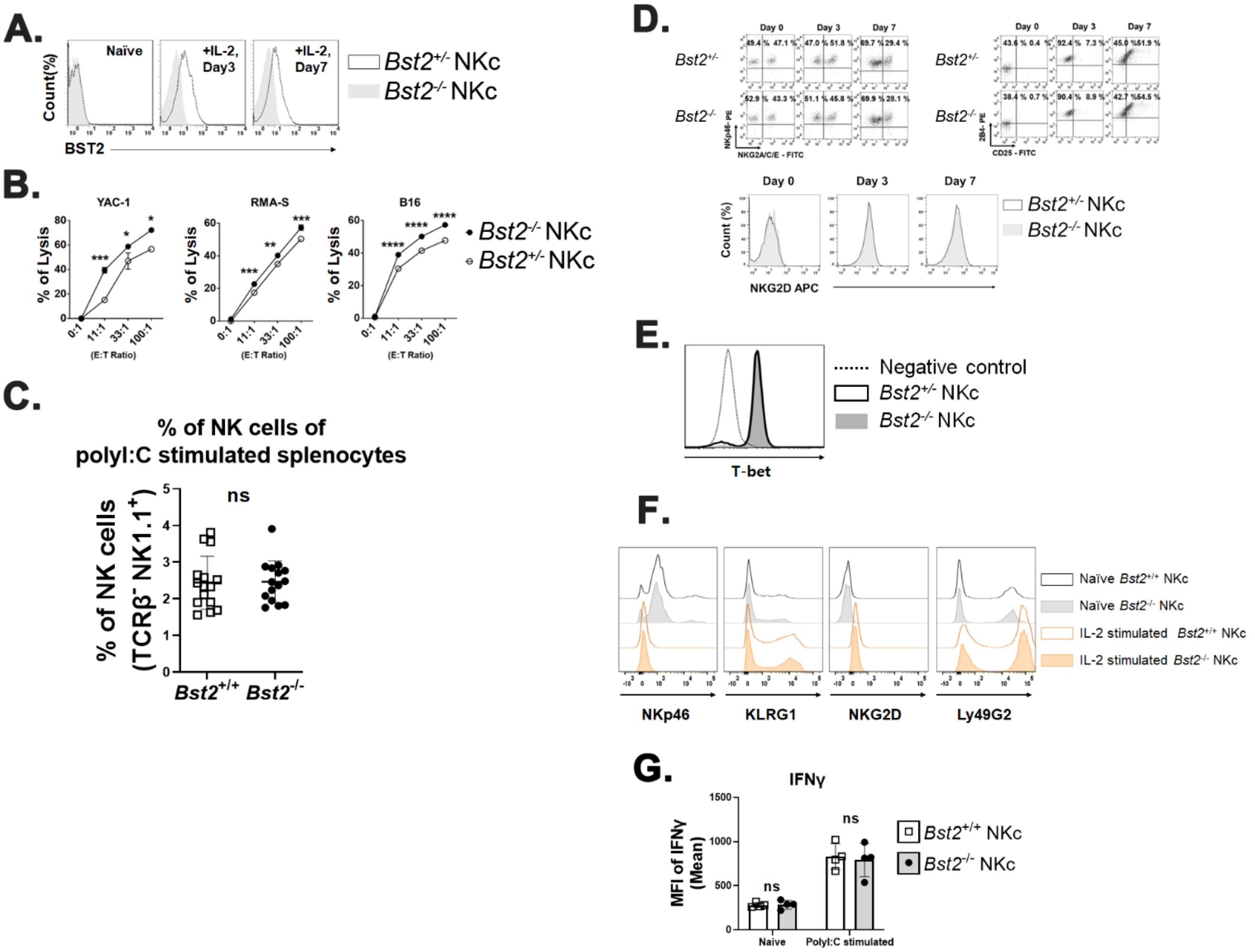

Figure S2. *Bst2*$^{-/-}$ NK cells have higher cytotoxicity to tumor cells than *Bst2*$^{+/-}$ NK cells. Naïve splenocytes of B6-origin *Bst2*$^{+/-}$ and *Bst2*$^{-/-}$ mice were stimulated by IL-2 treatment for seven days to generate LAK cells. (A) *Bst2*$^{+/-}$ and *Bst2*$^{-/-}$ LAK cells were stained with anti-BST2 antibody and analyzed by flow cytometry. (B) CFSE stained *Bst2*$^{+/-}$ and *Bst2*$^{-/-}$ LAK cells were co-cultured with target cells (YAC-1, RMA-S and B16) for 4 hrs. Cells were stained with 7-AAD after cultivation. Cells were then fixed with 1% paraformaldehyde and analyzed by flow cytometry. CFSE^{-} 7-AAD^{+} population was designated as lysed target cells. (C) B6-origin *Bst2*$^{+/+}$ and *Bst2*$^{-/-}$ mice were intraperitoneally injected with polyI:C. At 16 hrs after injections, whole splenocytes were analyzed by flow cytometry. The percentage of NK1.1^{+}TCRβ^{-} cells were considered as NK cells. (D, E) To compare the status of *Bst2*$^{+/-}$ and *Bst2*$^{-/-}$ LAK cells, NKp46, NKG2A/C/E, 2B4, CD25, NKG2D and transcription factor T-bet on LAK cells were analyzed through flow cytometry. (F) To compare the status of *Bst2*$^{+/+}$ and *Bst2*$^{-/-}$ LAK cells, NKp46, KLRG1, NKG2D and Ly49G2 on LAK cells were analyzed through flow cytometry. (G) DX5^{+} sorted Naïve NK cells were stimulated with polyI:C and IL-2 for 16 hrs in vitro. Mean fluorescence intensity (MFI) of IFN-γ of NK cells was analyzed by flow cytometry. (*, $p < 0.05$; **, $p < 0.01$; ***, $p < 0.001$; ****, $p < 0.0001$; n.s., not significant).

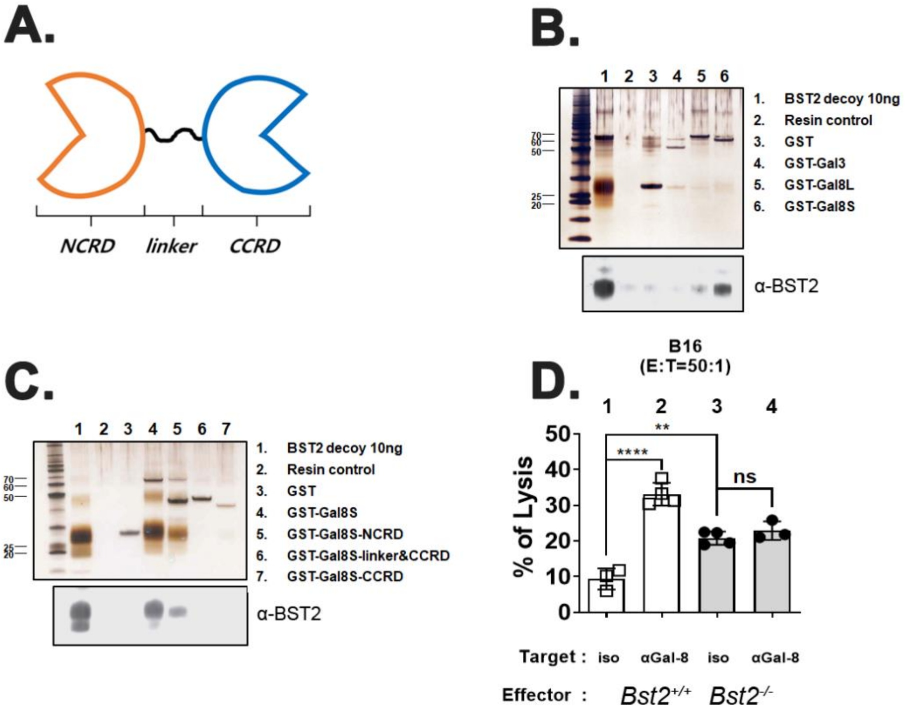

Figure S3. NCRDs of galectin-8 and galectin 9 are bound to BST2.(A) Schematic structure of tandem-repeat type galectins. (B) Recombinant BST2 protein was pulled down with GST-tagged Gal-3(GST-Gal3) as well as GST-tagged long isoform of Gal-8 (GST-Gal8L) and short isoform of Gal-8 (GST-Gal8S). Precipitates were analyzed by western blotting with anti-BST-2 antibody. (C) Recombinant BST2 protein was pulled down with GST-Gal8 as well as the N-terminal carbohydrate recognition domain of GST-tagged short isoform of Gal-8 (GST-Gal8S-NCRD) or C-terminal carbohydrate recognition domain of GST-tagged short isoform of Gal-8 (GST-Gal8S-CCRD) or linker conjugated GST-Gal8S-CCRD (GST-Gal8S-linker&CCRD). Precipitates were then analyzed by western blotting with anti-BST-2 antibody. (D) Naïve splenocytes of B6-origin *Bst2*$^{+/+}$ and *Bst2*$^{-/-}$ mice were stimulated by IL-2 treatment for seven days to generate LAK cells. CFSE stained *Bst2*$^{+/+}$ and *Bst2*$^{-/-}$ LAK cells were co-cultured with target cells (B16) for 4 hrs at 37°C in a humidified incubator in the presence of isotype control antibody or anti-Gal-8 antibody. Cells were stained with 7-AAD after cultivation. Cells were then fixed with 1% paraformaldehyde and analyzed by flow cytometry. CFSE^{-} 7-AAD^{+} population was designated as lysed target cells. (**, $p < 0.01$; ****, $p < 0.0001$; n.s., not significant).

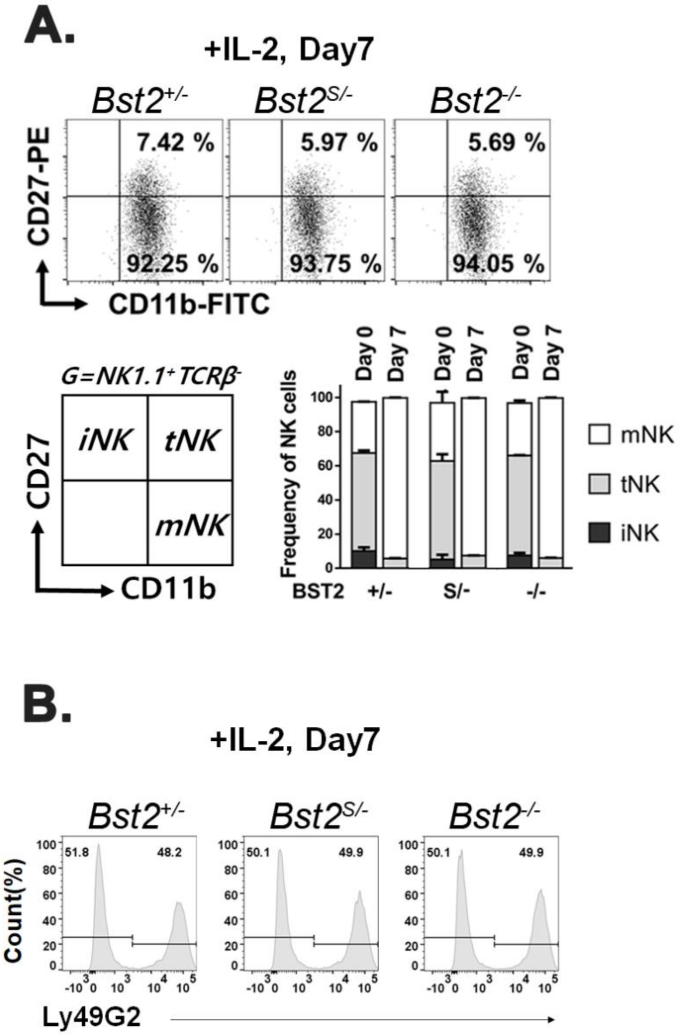

Figure S4. NK status markers are independent of BST2 expression. (A) LAK cells at day 0 and day 7 were stained with anti-CD27 and anti-CD11b antibodies. Developmental stages of NK cells were indicated. Immature NK cell (iNK) as $CD11b^{-}CD27^{+}$; Transit NK cell(tNK) as $CD11b^{+}CD27^{+}$; Mature NK (mNK) as $CD11b^{+}CD27^{-}$. (B) LAK cells at day 7 were stained with anti-Ly49G2 antibody. (B) The same cells were stained with anti-Ly49G2 antibody.

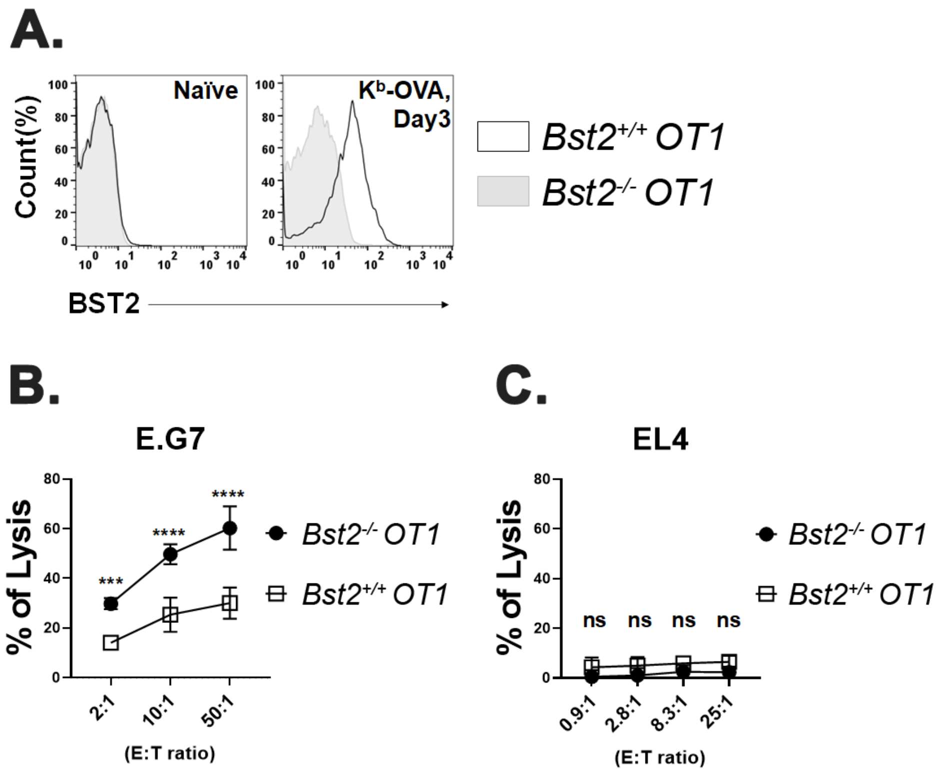

Figure S5. *Bst2*$^{-/-}$ OT1 cells have higher cytotoxicity to antigen-specific target cells than *Bst2*$^{+/+}$ OT1 cells. Naïve OT1 cells were sorted from B6-origin *Bst2*$^{+/+}$ OT1 Tg and *Bst2*$^{-/-}$ OT1 Tg mice. OT1 cells were stimulated by K^{b}-OVA/anti-CD28 beads and IL-2 treatment for three days. (A) *Bst2*$^{+/+}$ and *Bst2*$^{-/-}$ OT1 cells were stained with anti-BST2 antibody and analyzed by flow cytometry. (B, C) Calcein-AM stained target cells (E.G7-OVA and EL4) were co-cultured with *Bst2*$^{+/+}$ and *Bst2*$^{-/-}$ OT1 cells for 4 hr at 37°C in a humidified incubator. Released calcein was measured by SpectraMAX with excitation and emission wavelengths of 485 nm and 535 nm, respectively. (***, $p < 0.001$; ****, $p < 0.0001$; n.s., not significant).
